# Identification and mutational analyses of phosphorylation sites of the calcineurin-binding protein CbpA and the identification of domains required for calcineurin binding in *Aspergillus fumigatus*

**DOI:** 10.3389/fmicb.2015.00175

**Published:** 2015-03-13

**Authors:** Praveen R. Juvvadi, Yan Ma, Amber D. Richards, Erik J. Soderblom, M. Arthur Moseley, Frédéric Lamoth, William J. Steinbach

**Affiliations:** ^1^Division of Pediatric Infectious Diseases, Department of Pediatrics, Duke University Medical CenterDurham, NC, USA; ^2^Department of Dermatology and Venereology, The Second Hospital of Shanxi Medical UniversityTaiyuan, Shanxi, China; ^3^Duke Proteomics and Metabolomics Core Facility, Center for Genomic and Computational Biology, Duke UniversityDurham, NC, USA; ^4^Infectious Diseases Service, Department of Medicine, Lausanne University HospitalLausanne, Switzerland; ^5^Institute of Microbiology, Lausanne University HospitalLausanne, Switzerland; ^6^Department of Molecular Genetics and Microbiology, Duke University Medical CenterDurham, NC, USA

**Keywords:** *Aspergillus fumigatus*, calcineurin, calcineurin-binding protein (CbpA), phosphorylation, mutation

## Abstract

Calcineurin is a key protein phosphatase required for hyphal growth and virulence in *Aspergillus fumigatus*, making it an attractive antifungal target. However, currently available calcineurin inhibitors, FK506 and cyclosporine A, are immunosuppressive, limiting usage in the treatment of patients with invasive aspergillosis. Therefore, the identification of endogenous inhibitors of calcineurin belonging to the calcipressin family is an important parallel strategy. We previously identified the gene *cbpA* as the *A. fumigatus* calcipressin member and showed its involvement in hyphal growth and calcium homeostasis. However, the mechanism of its activation/inhibition through phosphorylation and its interaction with calcineurin remains unknown. Here we show that *A. fumigatus* CbpA is phosphorylated at three distinct domains, including the conserved SP repeat motif (phosphorylated domain-I; PD-I), a filamentous fungal-specific domain (PD-II), and the C-terminal CIC motif (Calcipressin Inhibitor of Calcineurin; PD-III). While mutation of three phosphorylated residues (Ser208, Ser217, Ser223) in the PD-II did not affect CbpA function *in vivo,* mutation of the two phosphorylated serines (Ser156, Ser160) in the SP repeat motif caused reduced hyphal growth and sensitivity to oxidative stress. Mutational analysis in the key domains in calcineurin A (CnaA) and proteomic interaction studies confirmed the requirement of PxIxIT motif-binding residues (352-NIR-354) and the calcineurin B (CnaB)-binding helix residue (V371) for the binding of CbpA to CnaA. Additionally, while the calmodulin-binding residues (442-RVF-444) did not affect CbpA binding to CnaA, three mutations (T359P, H361L, and L365S) clustered between the CnaA catalytic and the CnaB-binding helix were also required for CbpA binding. This is the first study to analyze the phosphorylation status of calcipressin in filamentous fungi and identify the domains required for binding to calcineurin.

## Introduction

Calcineurin (also known as protein phosphatase 2B) is an important protein phosphatase essential for hyphal growth, development, and virulence in *Aspergillus fumigatus*, making it an attractive antifungal target (Steinbach et al., 2007). However, due to direct immunosuppressive effects, administration of currently available calcineurin inhibitors, FK506 and cyclosporine A, for the treatment of patients with invasive aspergillosis is not practical (Ho et al., 1996). Therefore, identification of endogenous inhibitors of calcineurin is important to more effectively inhibit calcineurin as part of future drug targeting strategies. One such group of endogenous inhibitors are the regulators of calcineurin (RCANs; RCAN1-3) or modulatory calcineurin-interacting proteins (MCIPs; MCIP1-3) belonging to the calcipressin family of proteins (Rothermel et al., 2003). This family is composed of key regulators of calcineurin-NFAT signaling in diverse organisms, ranging from the yeasts to humans (Kingsbury and Cunningham, 2000; Rothermel et al., 2003). Another group of scaffolding proteins, Cain/Cabin-1 and A-kinase anchoring protein 79 (AKAP79), have also been identified to interact and inhibit calcineurin function in a phosphorylation-dependent manner in mammalian cells (Coghlan et al., 1995; Kashishian et al., 1998; Lai et al., 1998). Despite the conservation of calcineurin from the yeasts to human, the fungal homologs of the endogenous mammalian calcineurin inhibitor Cabin 1/Cain have not yet been identified.

How RCAN proteins precisely modulate calcineurin function is debatable. Earlier reports in mammalian cells revealed that RCAN1, one of the target genes of NFAT, binds to calcineurin and inhibits its activity (Fuentes et al., 2000; Rothermel et al., 2000), but more recent reports suggest that RCAN1 actually facilitates the activation of the calcineurin-NFAT signaling pathway (Sanna et al., 2006). A study using a combination of *in silico* stimulations and single cell experiments indicated that RCAN1 inhibits calcineurin at lower concentrations, but functions as a facilitator when its levels increase within the cell (Shin et al., 2011).

Attempts to understand the molecular mechanism responsible for facilitation of calcineurin-NFAT signaling revealed that phosphorylation of RCAN1 switches it from an inhibitor to a facilitator (Shin et al., 2011). Earlier structural analysis of domains in RCAN1 revealed the requirement of PxIxIT and LxVP motifs in order to cause an inhibitory effect, and TxxP and ExxP motifs and a GSK3β phosphorylation site to act as a facilitator (Mehta et al., 2009). The hallmark of the members of the calcipressin family is the presence of a conserved serine-proline repeat (SP repeat; FxISPPxSPP motif) which is phosphorylated at the two serine residues by mitogen-activated kinase (MAPK), followed by glycogen synthase kinase 3 (GSK3β; Vega et al., 2002). Studies in *Saccharomyces cerevisiae* have shown that phosphorylation by GSK3β within the SP repeat is required for calcineurin activity (Hilioti et al., 2004). Previously, this GSK3β phosphorylation was shown to be reversed by the phosphatase action of calcineurin (Vega et al., 2002).

In the yeast *S. cerevisiae,* deletion of RCAN1 caused sensitivity to cation stress, as opposed to no cation sensitivity with deletion of *CBP1* (the RCAN1 ortholog) in *Cryptococcus neoformans* (Görlach et al., 2000). In the presence of the FK506-FKBP12 complex, binding of CBP1 to calcineurin was also inhibited in *C. neoformans* (Görlach et al., 2000). In addition to the coordinating role of Cbp1 with calcineurin in filamentation of *C. neoformans* during mating, the importance of phosphorylation and dephosphorylation of two serine residues in the SP repeat motif for directing activity of calcineurin during hyphal elongation was demonstrated through site-directed mutagenesis (Fox and Heitman, 2005). In *S. cerevisiae,* phosphorylation of RCAN1 promoted its degradation and decreased its inhibitory effect on calcineurin (Kishi et al., 2007).

We previously demonstrated the importance of the RCAN1 ortholog, CbpA, for hyphal growth and calcium homeostasis in *A. fumigatus* (Pinchai et al., 2009). While deletion of *cbpA* resulted in increased expression of the *vcxA* (vacuolar Ca^2+^/H^+^ exchanger), *chsA* (chitin synthase A), and *cnaA* genes, it only slightly attenuated virulence. Although we showed that *cbpA* deletion or overexpression altered *cnaA* transcriptional response, the biochemical mechanisms underlying *A. fumigatus* CbpA phosphorylation and the mechanism of how it binds to calcineurin remain unknown. In this study, using phosphopeptide enrichment and tandem mass spectrometry we identified that CbpA is phosphorylated at three distinct domains, including a conserved SP repeat motif, a non-conserved filamentous fungal-specific domain, and a C-terminal region with low homology to yeast and human orthologs. Assessment of CbpA phosphorylation status in the absence of CnaA *in vivo* revealed the continual phosphorylation of the two serine residues (Ser156, Ser160) in the SP repeat motif and the two serine residues (Ser217, Ser223) in the filamentous fungal-specific domain, indicating the probable role for calcineurin in the dephosphorylation of these residues. While mutation of the phosphorylated residues in the non-conserved filamentous fungal-specific domain did not result in any growth defects, mutations in the SP repeat motif caused decreased hyphal growth and caused sensitivity to oxidative stress. Furthermore, although we could not identify a consensus PxIxIT motif in *A. fumigatus* CbpA, we found the absolute requirement of PxIxIT motif-binding residues (352-NIR-354) and the calcineurin B (CnaB)-binding helix residue (V371) for the binding of CbpA to CnaA. The binding of CbpA to CnaA was not inhibited by mutation of the calmodulin-binding residues (442-RVF-444) in CnaA, indicating that CbpA binds to calcineurin independent of calmodulin. We also identified three residues (T359, H361, and L365) that are clustered between the catalytic and the CnaB-binding helix that are required for CbpA binding.

## Materials and Methods

### Strains and Culture Conditions

*Aspergillus fumigatus* strain *akuB^KU80^* and the isogenic *pyrG* auxotrophic strain *akuB*^KU80^ *pyrG*^-^ were used in all experiments. *A. fumigatus* cultures were grown on glucose minimal medium (GMM) at 37^°^C. *Escherichia coli* DH5α competent cells were used for subcloning. *A. fumigatus* was transformed as described earlier (Steinbach et al., 2006). For radial growth analyses, 10^4^ conidia of the *akuB^KU80^* and the other strains were cultured on GMM agar at 37^°^C, with colony diameters determined every 24 h over a period of 5 days. All growth experiments were performed in triplicate.

### Generation of *cbpA* Deletion and CbpA-EGFP Expression Strains

While a Δ*cbpA* strain was previously reported by our group (Pinchai et al., 2009), we generated a second deletion strain in the *akuB^KU80^* background for this study in order to maintain the same genetic background for all mutated strains. Deletion of *cbpA* was performed using a previously designed construct in the plasmid pJW24 with the *Aspergillus parasiticus pyrG* gene as the auxotrophic marker (Steinbach et al., 2006). The *cbpA* deletion construct contained 1 kb promoter and 780 bp terminator flanking regions to facilitate homologous recombination. The ∼5 kb *cbpA* deletion construct was PCR-amplified using the primers cbpA-promo-KpnI-F and cbpA-term-HindIII-R (Supplementary Table S1) and transformed into the *A. fumigatus akuB*^KU80^ *pyrG*^-^ strain. Transformants selected in the absence of uracil/uridine (Steinbach et al., 2006) were screened by PCR (Supplementary Table S1) and Southern analyses to confirm deletion of the *cbpA* gene (Supplementary Figure S1).

In order to purify the CbpA protein for phosphorylation analyses, the *cbpA-*tagged* egfp* expression construct at the *cbpA* native locus was generated by a fusion PCR strategy. First, the 864 bp *cbpA* genomic DNA without the stop-codon and the 722 bp *egfp* gene were PCR-amplified separately. Next, the two PCR fragments were combined and used as templates to amplify the final fusion PCR product containing the *cbpA*-*egfp* fusion product, which was then digested with KpnI and NotI to clone at the KpnI-NotI sites in pUCGH (gift from Axel Brakhage) with the hygromycin B resistant gene (*hph*) as the selection marker. The cloned *cbpA* genomic DNA was sequenced to ensure accuracy (Primers listed in Supplementary Table S1). To facilitate homologous recombination, the 780 bp *cbpA* terminator region was cloned into the SbfI-HindIII sites in pUCGH (Langfelder et al., 2001). The final plasmid obtained was designated as pUCGH-CbpA, and the entire construct was linearized by KpnI-HindIII digestion to obtain a fragment of ∼6.7 kb. The linearized construct was then transformed into the *A. fumigatus akuB^KU80^* strain as previously described and transformants selected by resistance to hygromycin B. The transformants obtained were verified for homologous integration by PCR and also by fluorescent microscopy to visualize GFP fluorescence.

### Construction of *cbpA* Mutations

Site-directed mutation of phosphorylated residues in CbpA was performed using primers listed in Supplementary Table S1 and pUCGH-*cbpA* as a template. Briefly, the first PCR used complementary primers overlapping the regions to be mutated and the respective primers at the opposite ends to amplify two PCR fragments. Next, a fusion PCR was performed by using an equiproportional mixture of the two PCR fragments as a template and amplifying the mutated PCR fragment using primers at the opposite ends (Supplementary Table S1). Each fragment cloned into pUCGH was sequenced to confirm the respective mutation and then linearized for homologous integration. The linearized constructs were transformed into the *A. fumigatus akuB*^KU80^ strain as previously described and transformants selected by resistance to hygromycin B. The transformants obtained were verified for homologous integration by PCR and also by fluorescent microscopy to visualize GFP fluorescence.

### Protein Extraction and GFP-Trap^®^ Affinity Purification

The *A. fumigatus* strain expressing the *cbpA-egfp* fusion construct was grown in GMM liquid medium as shaking cultures for 24 h at 37^°^C. Cell extracts were prepared by homogenizing the mycelia using liquid nitrogen in buffer A (50 mM Tris/HCl, pH 7.5, 150 mM NaCl, 50 mM KCl, 0.01% Triton X-100, 1 mM PMSF, and 1:100 protease inhibitor cocktail). Total cell lysate was initially centrifuged at 5000 rpm to eliminate cell debris, and clarified by further centrifugation at 7000 rpm for 10 min at 4^°^C. The final supernatant fraction was collected and protein content determined by Bradford assay. Total protein was normalized to contain ∼10 mg protein in the sample before GFP-Trap affinity purification (Chromotek). GFP-Trap^®^ resin (35 μl) was equilibrated by washing three times in 500 μl ice-cold dilution buffer (10 mM Tris-HCl pH 7.5, 150 mM NaCl, 0.5 mM EDTA, 1 mM PMSF, 1:100 Protease Inhibitory Cocktail) according to the manufacturer instructions and resuspended in 100 μl ice cold dilution buffer. The GFP-Trap^®^ resin suspension was then mixed with total crude cell lysate containing ∼10 mg total protein and incubated at 4^°^C by gentle agitation for 2 h. The suspension was centrifuged at 2000 rpm for 10 min at 4^°^C and the pelleted GFP-Trap^®^ resin was washed once in 500 μl of ice-cold dilution buffer and then twice with 500 μl of wash buffer (10 mM Tris-HCl pH 7.5, 350 mM NaCl, 0.5 mM EDTA, 1 mM PMSF, 1:100 Protease Inhibitory Cocktail).

### Sample Preparation for Mass Spectrometry

Protein-bound GFP-Trap^®^ resins were washed three times with 200 μl of 50 mM ammonium bicarbonate, pH 8.0, and suspended in 30 μl 50 mM ammonium bicarbonate, pH 8.0, supplemented with 0.1% Rapigest SF surfactant (Waters Corp). Samples were reduced with 5 mM dithiothreitol for 30 min at 70^°^C and free sulfhydryls were alkylated with 10 mM iodoacetamide for 45 min at room temperature. Proteolytic digestion was accomplished by the addition of 500 ng sequencing grade trypsin (Promega) directly to the resin, with incubation at 37^°^C for 18 h. Supernatants were collected following a 2 min centrifugation at 1000 rpm, acidified to pH 2.5 with TFA, and incubated at 60^°^C for 1 h to hydrolyze the remaining Rapigest surfactant. Insoluble hydrolyzed surfactant was cleared by centrifugation at 15,000 rpm for 5 min. Ninety percent (by volume) of the sample was then removed for subsequent phosphopeptide analysis, and the remaining 10% (by volume) was subjected to an unbiased protein interaction analysis.

### Phosphopeptide Enrichment and LC-MS/MS Analysis

For the phosphopeptide analysis, samples were lyophilized to dryness using vacuum centrifugation and resuspended in 65 μl 80% acetonitrile, 1% TFA. Peptides were subjected to phosphopeptide enrichment using a 10 μl GL Sciences TiO2 Spin Tip and subsequently washed with 80% acetonitrile, 1% TFA. Peptides were eluted in 50 μl 20% acetonitrile, 5% aqueous ammonia, pH 10.5, and then acidified to pH 2.5 with formic acid prior to lyophilization to dryness.

Samples were resuspended in 10 μl 2% acetonitrile, 10 mM citric acid 0.1% formic acid and subjected to chromatographic separation on a Waters NanoAquity UPLC equipped with a 1.7 μm HSS T3 C18 75 μm I.D. x250 mm reversed-phase column. Phosphopeptide enriched samples were additionally supplemented with 10 mM citric acid. The mobile phase consisted of (A) 0.1% formic acid in water and (B) 0.1% formic acid in acetonitrile. Following a 5 μl injection, peptides were trapped for 5 min on a 5 μm Symmetry C18 180 lm I.D. x20 mm column at 20 μl/min in 99.9% A.

The analytical column was connected to a fused silica PicoTip emitter (New Objective, Cambridge, MA, USA) with a 10 μm tip orifice. Non-phosphopeptide enriched samples were analyzed on a Synapt G2 QToF mass spectrometer operating in a data-dependent mode of acquisition with a precursor MS scan from m/z 400–2000 followed by three MS/MS scans at a CID energy of 30% and a dynamic exclusion of 30 s. Phosphopeptide enriched samples were analyzed on a QExactive Plus mass spectrometer with a precursor MS scan from m/z 300–1600 with *r* = 70,000 at m/z 200 and a target AGC setting of 1e6 ions. In a data-dependent mode of acquisition, MS/MS spectra of the 10 most abundant precursor ions were with a CID energy setting of 27 and a dynamic exclusion of 20 s was employed for previously fragmented precursor ions.

Raw LC-MS/MS data files were processed in Mascot distiller (Matrix Science) and then submitted to independent Mascot searches (Matrix Science) against a custom NCBI_*Aspergillus* database containing both forward and reverse entries of each protein. Search tolerances were 5 or 10 ppm for precursor ions and 0.02 or 0.04 Da for product ions using trypsin specificity with up to two missed cleavages for phosphopeptide enriched or non-phosphopeptide enriched data, respectively. Carbamidomethylation (+57.0214 Da on C) was set as a fixed modification, whereas oxidation (+15.9949 Da on M), deamidation (+0.98 Da on NQ), and phosphorylation (+79.98 Da on STY) were allowed. All searched spectra were imported into Scaffold (v4.0, Proteome Software) and scoring thresholds were set to achieve a protein false discovery rate of 0% using the PeptideProphet algorithm. Normalized spectral counts were used to estimate relative protein abundances for interaction studies. This was accomplished by adjusting the sum of the selected quantitative value for all proteins in the list within each MS sample to the average of the sums of all MS samples present in the experiment.

## Results and Discussion

### *Aspergillus fumigatus* CbpA is phosphorylated at Three Domains, Including the FxSPPxSPP Motif

As a first step toward identifying the phosphorylation status of CbpA *in vivo* we initially utilized the *A. fumigatus* strain expressing *cnaA*-*egfp* fusion construct considering the fact that CbpA would co-purify with CnaA. As shown in Supplementary Table S2, purification of CnaA by GFP-Trap^®^ affinity purification and phosphopeptide-enrichment (see Supplemental Materials and Methods) led to the identification of six phosphorylated residues in CbpA (Ser156, Ser160, Thr205, Ser208, Ser217, Ser223). Next, in order to validate this finding and more clearly verify the phosphorylation status of *A. fumigatus* CbpA *in vivo,* a strain expressing the *cbpA–egfp* fusion construct under the control of its native promoter was generated in the *akuB^KU80^* strain. The CbpA–EGFP fusion protein was purified by GFP-Trap^®^ affinity purification and subjected to protease digestion, followed by phosphopeptide-enrichment and tandem mass spectrometry. As shown in **Table 1**, a total of nine residues (Ser8, Thr133, Ser156, Ser160, Ser208, Ser217, Ser223, Ser257, and Ser266) were identified to be phosphorylated in *A. fumigatus* CbpA.

**Table 1:** List of phosphorylated peptides identified in CbpA by LC-MS/MS analysis.

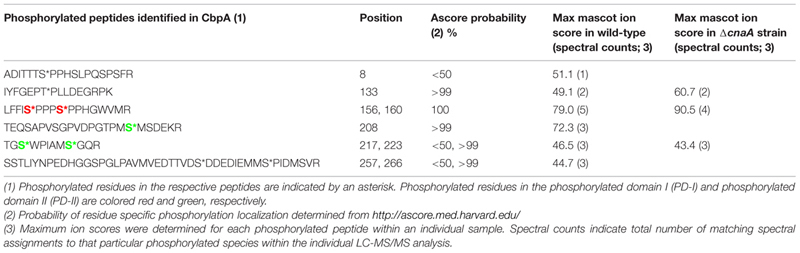

**FIGURE 1:**
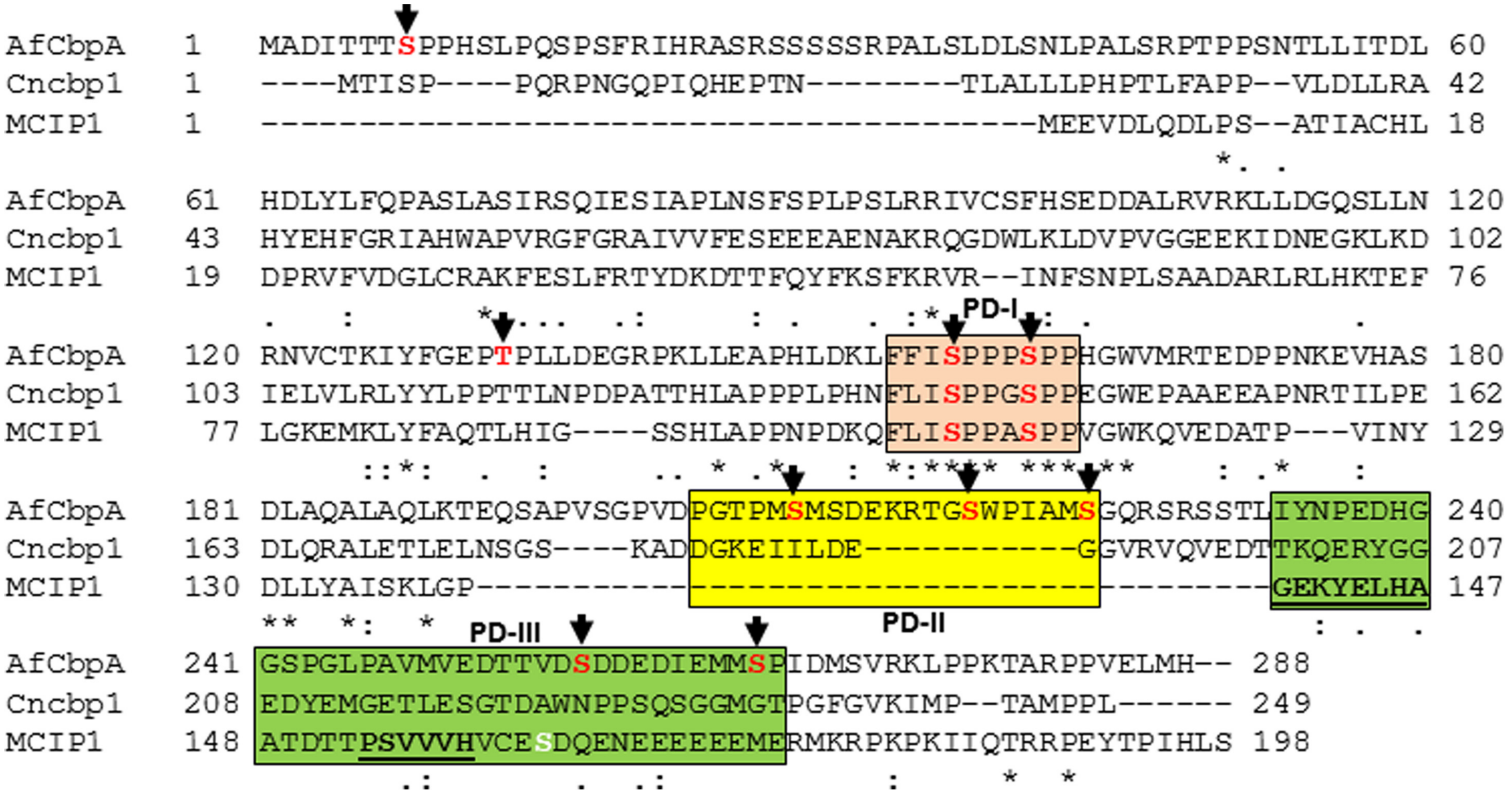
Clustal alignment of *Aspergillus fumigatus* CbpA (AfCbpA) with *Cryptococcus neoformans* Cbp1 (Cncbp1) and Human MCIP1. Conserved residues are indicated in asterisks. Arrows and residues shown in red indicate the identified phosphorylation sites in CbpA. The respective phosphorylated domains PD-I (in pink), PD-II (in yellow) and PD-III (in green) are boxed. The ELHA and the PxIxIT motifs (PSVVVH) in MCIP1 are underlined. The phosphorylated residue within the CIC-motif of Human MCIP1 (PD-III) is shown in white color.

Based on the location of the majority of the phosphorylated residues, they were classified into three different domains: phosphorylated domain I (PD-I), phosphorylated domain II (PD-II), and phosphorylated domain III (PD-III; **Figure 1**). While the phosphorylation of the FxISPPxSPP motif in the PD-I (Ser156, Ser160; Supplementary Figure S2) is well-known from mammalian and the yeast RCANs (Vega et al., 2002; Hilioti et al., 2004; Abbasi et al., 2006), the identification of three residues phosphorylated in PD-II (Ser208, Ser217, Ser223) seems to be specific to *A. fumigatus* as this region was not conserved in the *C. neoformans* Cbp1 or in the Human MCIP1 (**Figure 1**). In addition, phosphorylation of two serine residues (Ser257 and Ser266) in PD-III, previously designated as the CIC motif (Calcipressin Inhibitor of Calcineurin), was also noted. The calcineurin-binding PxIxIT sequence (PSVVVH) together with the ELHA sequence comprises the CIC motif and is conserved in all vertebrate RCANs/MCIPs (underlined in **Figure 1**; Aubareda et al., 2006; Mulero et al., 2007). Both the ELHA and PxIxIT sequences are required for binding to calcineurin with high affinity (Martínez-Martínez et al., 2009; Mulero et al., 2009). As shown in Supplementary Figure S3, the two serine residues (Ser156 and Ser160) in the FxISPPxSPP motif (PD-I) are conserved in all the filamentous fungi. The fungal orthologs, however, show less conservation to mammalian RCAN in the ELHA and PxIxIT domains (PD-III; Supplementary Figure S3). However, it is interesting to note that phosphorylation of a conserved serine residue was observed within the CIC (PD-III) motif of MCIP1 (**Figure 1**; serine residue shown in white color) and the protein kinase CK2 was identified as the enzyme responsible for the phosphorylation to potentiate inhibition of NFATc signaling by disrupting the calcineurin-NFATc interaction (Martínez-Høyer et al., 2013). Although we do not have conservation of the ELHA or the PSVVVH residues in the *A. fumigatus* CbpA CIC motif (PD-III), we do have the predicted CK2 target sequence (S/TxxE/D) within the PD-III.

While phosphorylation at the CIC motif (PD-III) was also suggested to be independent of the phosphorylation occurring at the FxISPPxSPP motif (PD-I) of the RCANs, the non-phosphorylated CIC (PD-III) caused increased NFATc (Martínez-Høyer et al., 2013). The absence of PD-II domain in the mammalian MCIP1, the non-conservation of the PD-III domain in the fungal orthologs (Supplementary Figure S3), and our recovery of PD-III phosphorylated CbpA from the wild-type strain all suggest possible differential regulation of the fungal Cbps through phosphorylation-dephosphorylation. In the future, it will be interesting to examine the effect of mutation of these residues on CbpA toward the CnaA–CrzA (ortholog of NFAT) interaction and activation.

### Deletion of CnaA Caused Reduction in the Phosphorylated Residues of CbpA

Previous studies have suggested the dephosphorylation of RCANs by calcineurin (Fox and Heitman, 2005). In the mammalian RCANs, phosphorylation of the two serine residues within the FxISPPxSPP motif (PD-I; Ser108 and Ser112) occurs first at Ser112 through MAPK, which then triggers subsequent phosphorylation at Ser108 by GSK3β (Vega et al., 2002). The phosphorylated Ser108 can then be dephosphorylated by calcineurin. It was also previously shown that alterations in the FxISPPxSPP motif (PD-I) phosphorylation influence protein stability, with mutation of the FxISPPxSPP leading to a more stable protein (Genesca et al., 2003). Genetic evidence from *S. cerevisiae* suggests that phosphorylation of Ser108 by GSK3 can release the inhibitory activity of RCAN and convert the protein to an activator of calcineurin, although the exact mechanism is unknown (Hilioti et al., 2004). Also it is unknown whether the phosphorylation at the CIC motif (PD-III) is subject to regulation by calcineurin protein phosphatase activity.

**FIGURE 2:**
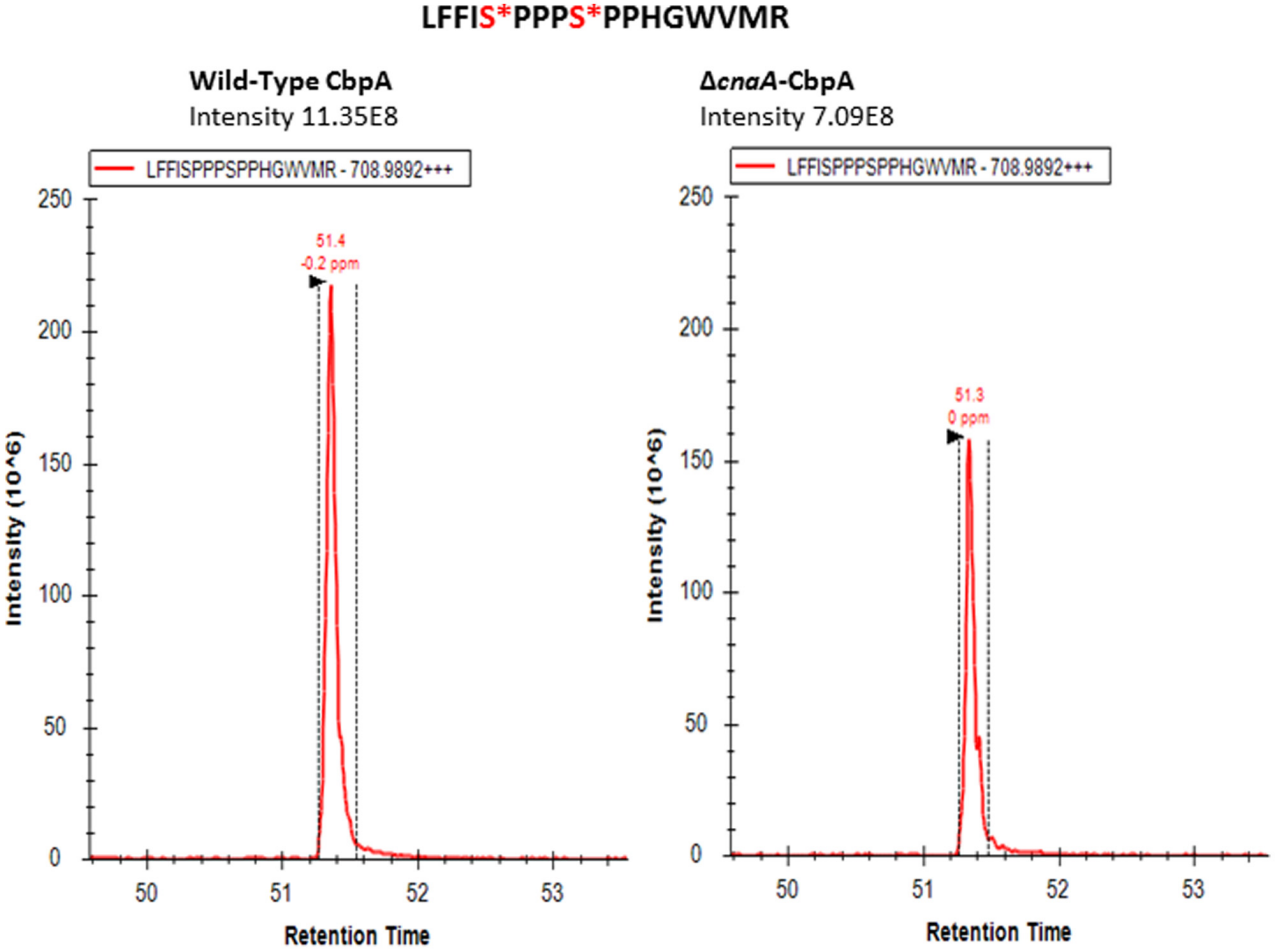
Selected ion chromatogram of LFFI[pS]PPP[pS]PPHGWVMR (m/z [708.9892]^2+^) from *A*. *fumigatus* CbpA in the *akuB^KU80^* (reference strain) and the Δ*cnaA* background strains.

Considering these data, and the rationale that absence of calcineurin may inhibit dephosphorylation of the residues that undergo calcineurin-mediated dephosphorylation, we next determined if any of the phosphorylated residues are targets for calcineurin by deleting *cnaA* and monitoring the phosphorylation status of CbpA. The FxISPPxSPP residues (PD-I; Ser156, Ser160; **Figure 2**) and Ser217 and Ser223 in the PD-II domain remained phosphorylated, in addition to the single Thr133 residue (**Table 1**). In addition to the N-terminal Ser8 residue, the Ser208 in the PD-II domain, and the residues in the CIC motif (PD-III; Ser257, Ser266) were not phosphorylated in the *cnaA* deletion background, indicating the possibility of calcineurin involvement in the phosphorylation-dephosphorylation mechanism of CbpA. Specifically, the residues in the FxISPPxSPP motif (PD-I) and the PD-II domain may be amenable for dephosphorylation by calcineurin.

### Phosphorylation and Dephosphorylation of CbpA at the FxSPPxSPP Motif (PD-I) is Important for Proper Hyphal Growth and Tolerance to Oxidative Stress

**FIGURE 3:**
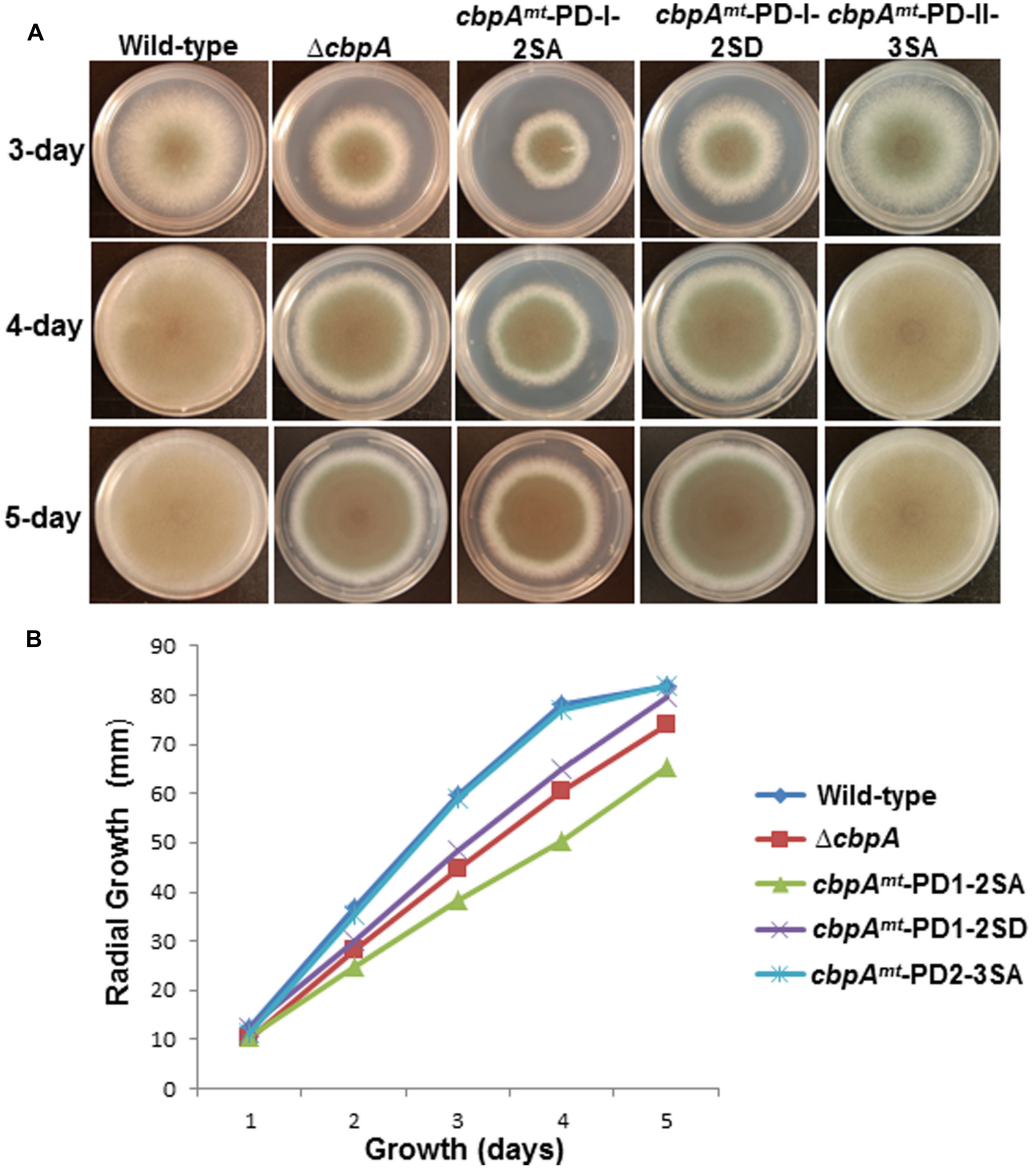
**(A)** Radial growth of the reference strain (wild-type; *akuB*^KU80^), the Δ*cbpA* strain, and the respective CbpA phosphorylation mutation strains (*cbpA^*mt*^*-PD-I-2SA; *cbpA^*mt*^*-PD-I-2SD and *cbpA^*mt*^*-PD-II-3SA) was assessed over a period of 5-days at 37^°^C on GMM agar medium. Growth of the strains at 3, 4, and 5 days are shown. A total of 1 × 10^4^ conidia were spotted for each strain. **(B)** Measurement of colony diameter of the strains over a period of 5 days. For radial growth quantification the strains were grown on GMM agar medium in triplicate and values are depicted as average colony diameter.

Phosphorylation of the FxISPPxSPP motif (PD-I) at both serine residues in *C. neoformans* Cbp1 was shown to be necessary for its stability, activity, and regulation of hyphal elongation during mating (Fox and Heitman, 2005). Interestingly, the phosphorylation of the FxISPPxSPP motif (PD-I) did not mediate the binding of calcineurin to Cbp1 (Fox and Heitman, 2005). In order to investigate the importance of phosphorylation of *A. fumigatus* CbpA at the FxISPPxSPP motif (PD-I), we next mutated the two phosphorylated residues within the FxISPPxSPP motif to alanine and aspartic acid residues, respectively, to mimic a non-phosphorylated state (*cbpA^*mt*^*-PD-I-2SA) and a constitutively phosphorylated state (*cbpA^*mt*^*-PD-I-2SD). Radial growth analyses of the strains under normal growth conditions over a period of 5 days indicated a slower growth rate in the *cbpA^mt^*-PD-I-2SA strain compared to the *cbpA^*mt*^*-PD-I-2SD strain (**Figure 3**). Unexpectedly, the Δ*cbpA* strain displayed a slightly better growth rate than the *cbpA^*mt*^*-PD-I-2SA strain. Based on our previous report (Pinchai et al., 2009), it is possible that the complete deletion of *cbpA* induces higher expression of calcineurin or inappropriate activation of calcineurin that can negatively affect growth. Although the *cbpA^*mt*^*-PD-I-2SD strain grew better than the *cbpA^*mt*^*-PD-I-2SA strain, its growth was comparable to the Δ*cbpA* strain, indicating that both dephosphorylation and continued phosphorylation of CbpA at the two serine residues in the FxISPPxSPP motif (PD-I) affected its activity. A similar observation was made with *C. neoformans* Cbp1-phospho mimetic expression, wherein mutation of both the serines to glutamic acid abolished filamentation during mating, a phenotype also observed with complete deletion of Cbp1 (Görlach et al., 2000). Importantly, in contrast to the FxISPPxSPP motif mutations (PD-I), the mutation of the three phosphorylated residues in the PD-II domain did not have any effect on the growth under normal conditions (**Figure 1**).

**FIGURE 4:**
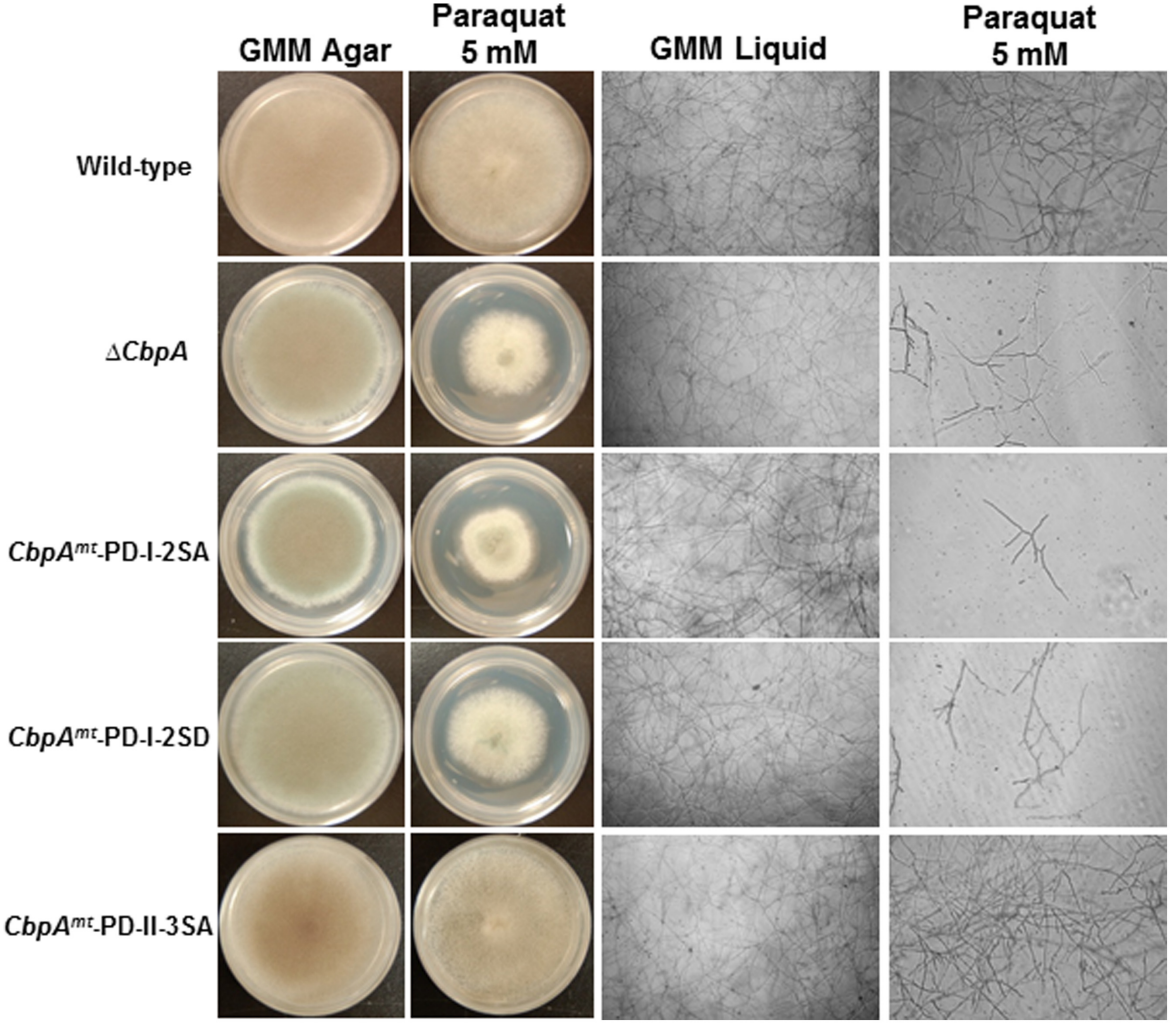
The reference strain (wild-type; *akuB*^**KU80**^), the Δ*cbpA* strain, and the respective CbpA phosphorylation mutation strains (*cbpA^**mt**^*-PD-I-2SA; *cbpA^**mt**^*-PD-I-2SD and *cbpA^**mt**^*-PD-II-3SA) were cultured in the absence or presence of the oxidative stress inducer paraquat (5 mM). Strains were grown for a period of 5-days on GMM agar supplemented with the respective oxidative stress inducers at 37^°^C. To more clearly distinguish the susceptibility of the strains to oxidative stress agents the respective strains were cultured in GMM liquid medium supplemented with paraquat (5 mM) and light-microscopy photographs (×10 magnification) were taken after 48 h of growth at 37^°^C. For liquid growth assays 1 × 10^4^ conidia of each strain were inoculated into 200 μl of GMM in multi-well plate.

In mammalian cells, increased levels of RCAN1 were found to be protective against oxidative stress and suppress cell growth. It is not known whether these features of RCANs are a direct consequence of calcineurin inhibition or result from a yet unidentified action of RCAN (Leahy and Crawford, 2000). Furthermore, phosphorylation of the mammalian RCANs at the FxISPPxSPP motif (PD-I) was correlated with an increase in calcineurin-mediated transcriptional inhibition (Genesca et al., 2003). Hence, in order to determine the role of phosphorylated CbpA in counteracting oxidative stress, the respective *cbpA* mutant strains were assayed for growth in the presence of the oxidative stress inducer paraquat (**Figure 4**). Based on phenotypes observed under normal growth conditions, phosphorylation of CbpA at the FxISPPxSPP motif (PD-I) is important for proper hyphal growth, but the phosphorylation at the PD-II domain seemed unimportant for growth. Furthermore, both the *cbpA^*mt*^*-PD-I-2SA and the *cbpA^*mt*^*-PD-I-2SD strains were sensitive to oxidative stress induced by paraquat (**Figure 4**) and H_2_O_2_ (data not shown), indicating the importance of phosphorylation-dephosphorylation of CbpA for counteracting oxidative stress conditions. The possibility of other phosphatases regulating the dephosphorylation of CbpA cannot be precluded.

### CnaA Requires the PxIxIT-Binding Motif and CnaB-Binding Helix But not the CaM-Binding Helix for Binding to CbpA

It was previously demonstrated that binding of mammalian RCANs to the calcineurin catalytic subunit (CnA) does not interfere with binding of either calmodulin (CaM) or the calcineurin regulatory subunit (CnB) to CnA (Vega et al., 2002). In fact, most data indicate that RCANs bind preferentially to active calcineurin, suggesting that CaM binding facilitates RCAN binding (Martínez-Martínez et al., 2009). However, in *C. neoformans* it was shown that the regulatory subunit of calcineurin is required for the binding of CBP1 to drugs that inhibit calcineurin activity (cyclosporin A or FK506) and also disrupt the CBP1/CNA interaction (Görlach et al., 2000), suggesting that the site of CBP1 interaction with CNA overlaps with that of immunophilins.

We previously generated *A. fumigatus* strains harboring mutations in key domains of CnaA (the PxIxIT-binding motif; CnaB binding helix; CaM-binding domain) to understand their significance for calcineurin localization and function (Juvvadi et al., 2013). All the respective *cnaA* mutated constructs were tagged to *egfp* and verified for stable expression (Juvvadi et al., 2013). Because there is no predictable PxIxIT motif in the *A. fumigatus* CbpA, we utilized the *A. fumigatus* strain expressing CnaA-NIR^mt^-AAA in which the PxIxIT motif-binding residues (352-NIR-354; Asn352 Ile353 Arg354) are mutated to alanines in CnaA to determine if the binding of CbpA to CnaA requires the PxIxIT motif. As shown in **Table 2**, we found that mutation of the PxIxIT motif-binding NIR residues in CnaA completely abolished the binding of CbpA to CnaA. The binding of CbpA to CnaA was also found to be dependent on CnaB binding to CnaA. This was demonstrated by utilizing the *A. fumigatus* mutant strains expressing CnaA^mt^-V371D and CnaA-THL^mt^-PLS constructs wherein the key CnaB-binding residue Val371 on CnaA is mutated to aspartic acid (V371D) and three other residues (THL^mt^-PLS; Thr359 His361 Leu365 mutated to Pro359 Leu361 Ser361, respectively) located close to the CnaB binding helix completely inhibited the binding of CbpA to CnaA. Moreover, treatment with FK506 also inhibited the binding of CnaA to CbpA. Contrary to these findings, mutation of the CaM binding domain residues RVF (442-RVF-444; Arg442 Val443 Phe444 to alanines) on CnaA did not affect the binding of CbpA to CnaA. The strain expressing wild-type CnaA-EGFP was used as a comparative control to determine the CbpA peptides bound to CnaA. The binding of CbpA peptides to CnaA were validated in three independent experiments by mass spectrometry (**Table 2** and Supplementary Table S3). Taken together, these results indicated that CbpA binds to CnaA through a PxIxIT motif and requires the binding of CnaB to CnaA. Although we could not identify the PxIxIT motif in CbpA, it is possible that a PxIxIT-like sequence (PVDPGT) close to the newly identified phosphorylated domain II (PD II) is responsible for binding to CnaA. Future crystallization studies on the calcineurin-CbpA complex would shed light on the role of each of these domains and the functional motifs in the binding of CbpA to calcineurin. Furthermore, detailed knowledge of the molecular mechanisms governing the CbpA-calcineurin interaction may also prove useful in the rational design of future immunosuppressant drugs.

**Table 2:** Binding analysis of CbpA to various mutated calcineurin A (CnaAs) *in vivo*.

Strains/treatment	Interacted with CnaA	Uniquely identified peptides (1)	Normalized total spectral counts (2)
Wild type	+	SQIESIAPLNSFSPLPSLR LLDGQSLLNR IYFGEPTPLLDEGRPK EVHASDLAQALAQL TGSWPIAMSGQR	7
Wild type + FK506	-	-	-
CnaA-NIR^mt^-AAA	-	-	-
CnaA-RVF^mt^-AAA	+	SQIESIAPLNSFSPLPSLR LLDGQSLLNR IYFGEPTPLLDEGRPK EVHASDLAQALAQL TGSWPIAMSGQR LLEAPHLDK	6
CnaA-V371D	-	-	-
CnaA-THL^mt^-PLS	-	-	-

*(1) Total number of unique peptide sequences identified to CbpA within the corresponding LC-MS/MS analysis*.

*(2) Normalized spectral counts reported from Scaffold following adjusting the sum of the selected quantitative value for all proteins in the list within each MS sample to a common value: the average of the sums of all MS samples present in the experiment*.

A recent study using human cell lines identified the phosphorylation of a serine residue at the CIC motif in all the RCANs (RCAN1, RCAN2, and RCAN3; Martínez-Høyer et al., 2013). Through *in vitro* and *in vivo* phosphorylation assays, CK2 was identified as the kinase phosphorylating this serine residue and this phosphorylation enhanced the disruptive potential toward the calcineurin-NFATc interaction. Interestingly, in the present study, the serine (Ser257) along with another serine residue at position 266 was identified to be phosphorylated the region spanning the CIC motif (PD-III) in *A. fumigatus* CbpA. In the future, it will be interesting to examine the effect of mutation of these residues on CbpA toward the CnaA-CrzA interaction and activation. Taken together, our results indicate that phosphorylation of CbpA at the FxISPPxSPP motif (SP repeat motif) is conserved in filamentous fungi and this phosphorylation is important for CbpA function *in vivo*. In addition, the binding of CbpA to CnaA occurs via a PxIxIT motif and the CnaB binding helix is essential for the interaction.

## Author Contributions

PJ and WS conceived and designed research; PJ, YM, AR, and FL performed research; PJ, ES, and MM acquired and analyzed the proteomics data; PJ and WS wrote the paper; PJ, YM, AR, ES, MM, FL, and WS approved the final submission.
