# Supplementary material for: Identification and mutational analyses of phosphorylation sites of the calcineurin-binding protein CbpA and the identification of domains required for calcineurin binding in Aspergillus fumigatus
Source: Front Microbiol. 2015 Mar 13;6:175. doi: 10.3389/fmicb.2015.00175 (PMC4358225; doi:10.3389/fmicb.2015.00175)
Supplement: Supplementary file 1 [file data_sheet_1.docx]

**A**


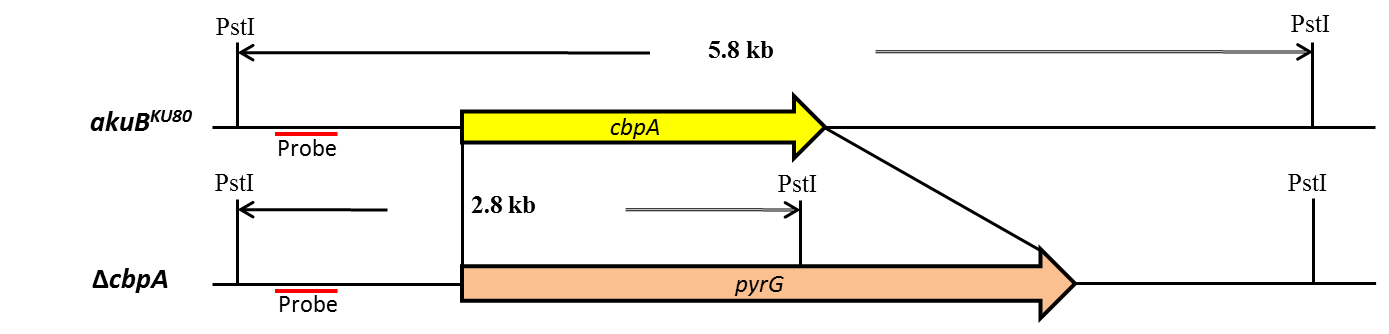


**Supplemental Figure S1**


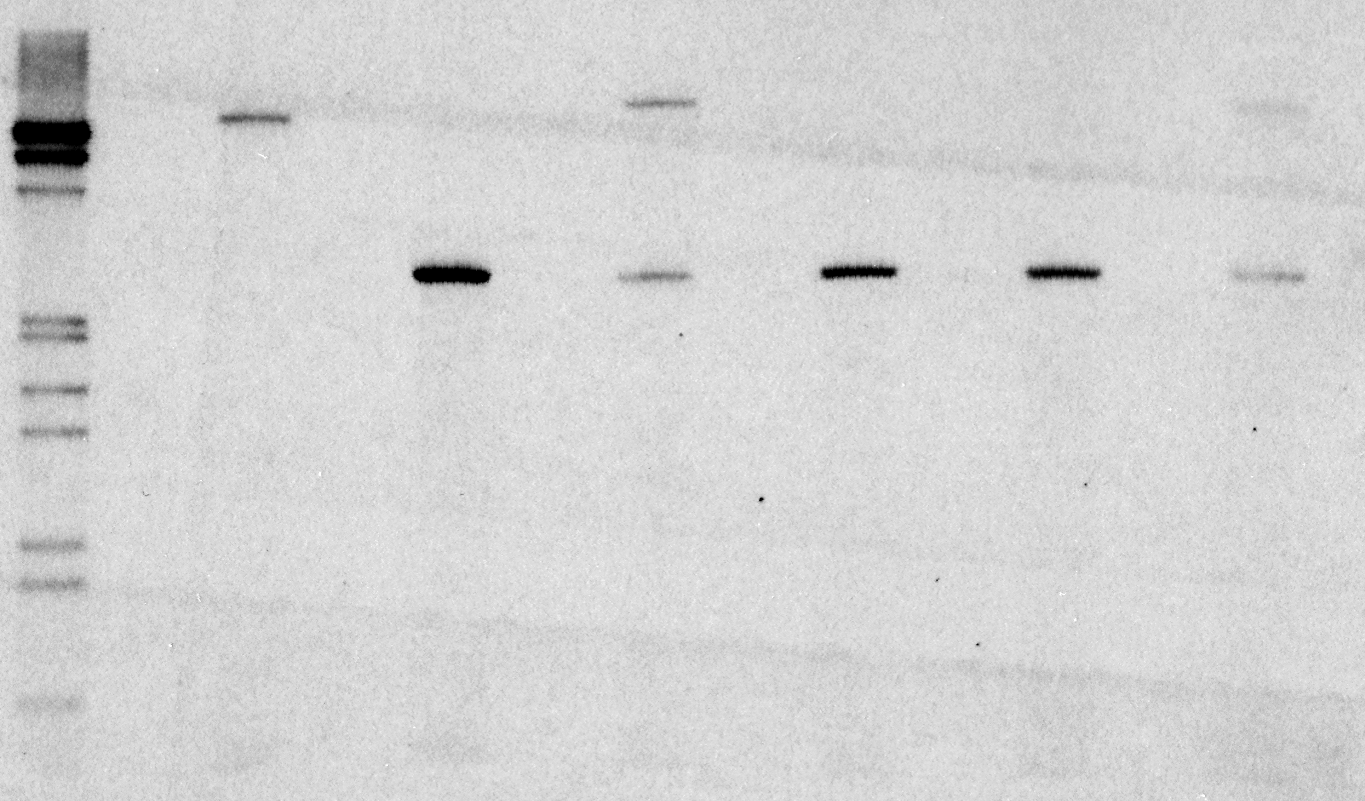


***akuB^KU80^* Δ*cbpA***

5771 bp

2817 bp

**DIG**

**marker (bp)**

2027

1904

3530

4268

5148

1584

1375

21226

831

947

564

**C**

**B**


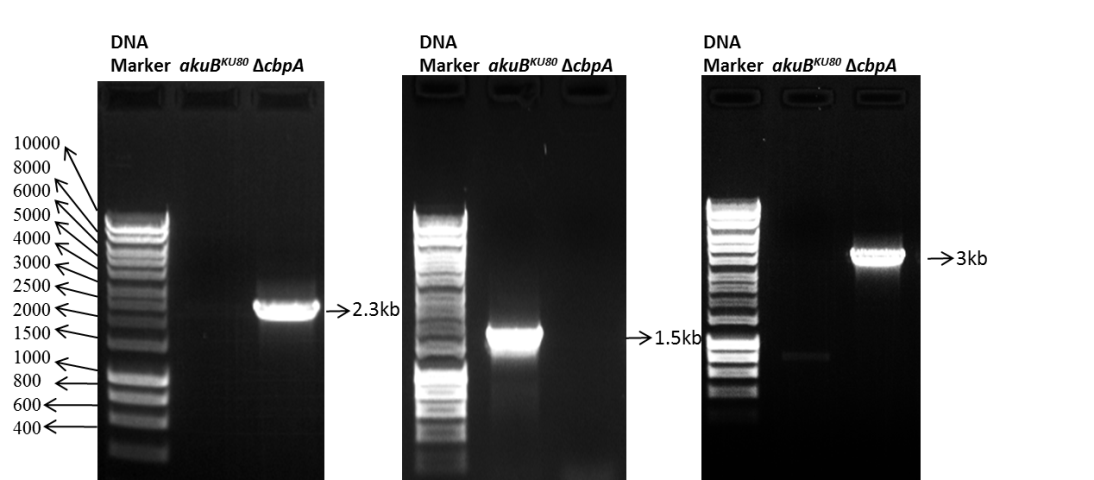


**(1)**

**(2)**

**(3)**

| **Figure B** | **Primers** | ***akuB^KU80^*** | **Δ*cbpA*** |
| --- | --- | --- | --- |
| (1) | 3’pyrG  5’pyrG | No PCR product | 2.3 kb |
| (2) | CbpA-KpnI-F  CbpA-term-HindIII-R | 1.5 kb | No PCR product |
| (3) | 5’pyrG  CbpA-term-HindIII-R | No PCR product | 3 kb |

**Fig. S1.** (A) Schematic representation of the genomic locus of the *akuB^KU80^* and the Δ*cbpA* deletion strains. The entire coding sequence of the *A. fumigatus* *cbpA* gene was replaced with the *A. parasiticus pyrG* gene by homologous recombination. Southern analysis with PstI-digested genomic DNA and *cbpA* left flank probe (indicated in red line) shows the replacement of *cbpA* by *pyrG* as a ~5.8 kb fragment in the ∆*cbpA* strain. (B) PCR confirmation of the *akuB^KU80^* and the Δ*cbpA* strains using different sets of primers as outlined in the table below (1, 2, 3). (C) Genomic DNA from both the *akuB^KU80^* and the Δ*cbpA* strain was digested with PstI; The DIG-labeled probe bound to a ~2.8 kb and a ~5.8 kb band in the *akuB^KU80^* and the Δ*cbpA* strain, respectively, showing the replacement of *cbpA* by *pyrG* as a ~5.8 kb fragment in the ∆*cbpA* strain.

**Supplemental Figure S2**


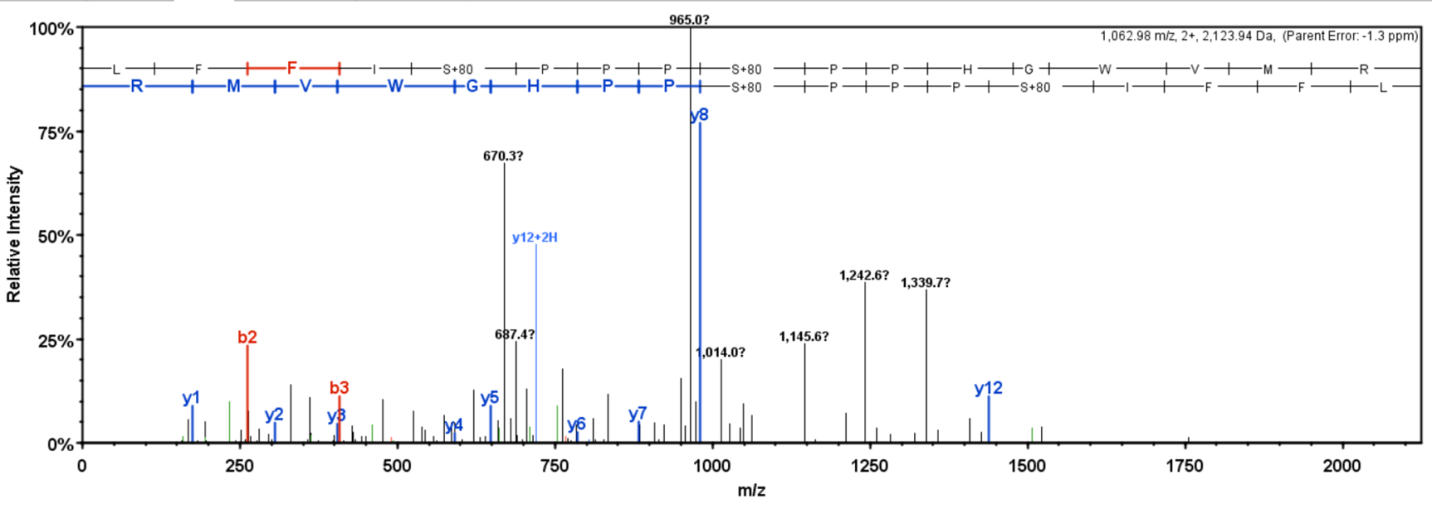


**Fig. S2.** Tandem mass spectrum of LFFI[pS]PPP[pS]PPHGWVMR (m/z [708.9892]^2+^) from *Aspergillus fumigatus* CbpA identified with a Mascot Ion Score of 79.0 and an AScore localization probability of 100%.

**Supplemental Figure S3**

MoCbpA -------------------MSQNDIRPSLPTRTSTSSSVSRRSNLSLDLSNLPPLTQPVT

FfCbpA -------------------------METSPQHSRSSSSASRRSNLTLDLSNIPPMTPPTP

NcCbpA MEPQQQQQQQQQVPSEQSLQQSPDLRPASLSRSSTGRSSRKGSSLSLDLSNLPPLSQPTP

AfCbpA ----------MADITTTSPPHSLPQSPSFRIHRASRSSSSSRPALSLDLSNLPALSRPTP

CnCbp1 --------------------------------------------MTISPPQRPNGQPIQH

Human-MCIP1 -------------------------------------------MEEVDLQDLP-------

ScRcn1 ------------------------------------------------MGNIITDTIIIT

:

MoCbpA PTNTLLFTNLLDRDIFRPDNLETIRNLVTKTAPIHSFAPLKSFARIVISFFSEEDAIAVR

FfCbpA PSNTLLFTNLTDPAIFLPENLQVIRDLITHSAPIHAFAPLKSFRRIVVSFFDEQAAIAVR

NcCbpA PSNTLIFTNINSLDVFSADNLQTIRDLISQTAPIFAWSPLKSFRRIIVTFFDEQAAIAVR

AfCbpA PSNTLLITDLHDLYLFQPASLASIRSQIESIAPLNSFSPLPSLRRIVCSFHSEDDALRVR

CnCbp1 EPTNTLALLLPHPTLFAPPVLDLLRAHYEHFGRIAHWAPVRGFGRAIVVFESEEEAENAK

Human-MCIP1 --SATIACHLDPRVFVDGLCRAKFESLFRTYDKDITFQYFKSFKRVRINFSNPFSAADAR

ScRcn1 SDKCDIVDNDNVERIQVWLSKNILRKFQINENEPLQLIILKRFKRILLICPSHDISQHVM

. : . :. . : * . : .

MoCbpA RVWDGEATMG------------------ASCNVYFGRPTPLDV--KEERLALPDAGKLFF

FfCbpA QVWDNEAIMG------------------QQCRVYFGMPTPVDK--RDEHLALPDAGKLFF

NcCbpA SVWDGEAILG------------------ERCRVYFGQPTPIDVSAADKHLALPDAGKLFF

AfCbpA KLLDGQSLLNRN----------------VCTKIYFGEPTPLLDEGRPKLLEAPHLDKLFF

CnCbp1 RQGDWLKLDVPVGGEEKIDNEGKLKDIELVLRLYYLPPTTLNPDPATTHLAPPPLPHNFL

Human-MCIP1 LQLHKTEFLG------------------KEMKLYFAQTLHIGS----SHLAPPNPDKQFL

ScRcn1 DASRALEMEN-------------------FNFSYSLQDGQRNLT--KQYLKVPESEKMFL

* * * : *:

**ELHA**

MoCbpA ISPPPSPPHDWEM-RLEDAPNKLVHAEDLAEALARLHHN---KDASGLSGVQTPISPTGG

FfCbpA ISPPPSPPHGWEM-RLEDAPNKLVHAEDLADALAKLHHRPGPMDEDQDSPVTPPDSALPG

NcCbpA ISPPPSPPHDWEQ-RMEDAPNTMVHAEDLAEALAKLRHHNNPNGIDADVKAPVSPASDGG

AfCbpA ISPPPSPPHGWVM-RTEDPPNKEVHASDLAQALAQLKTEQSAPVSGPVDPGTPMSMSDEK

CnCbp1 ISPPGSPPEGWEP-AAEEAPNRTILPEDLQRALETLELNSGSKADDGKEIILDEG-----

Human-MCIP1 ISPPASPPVGWKQ-VEDATP---VINYDLLYAISKLGPGEKYELHAATD-----------

ScRcn1 ISPPASPPPEFDFSKCEDAPQRHIQSHIQQDQQQRLEASQLLPNNPDKNN----------

**** *** : : .* : *

**PxIxIT**

MoCbpA ----------------NTRSRSSTLIFQPGQ---------DGASPELPAIAICDMTDEPE

FfCbpA ----------------RTRSRSSTLIYKP-----------EDAASTMPAVIVDDMTDEPE

NcCbpA SSSRPGAGGDFKEGARKNRSRSSTLIFQPEKKAQTSNGKANGPAVDLPCVTVDDMTDEAD

AfCbpA RTG------SWPIAMSGQRSRSSTLIYNPED---------HGGSPGLPAVMVEDTTVDSD

CnCbp1 ----------------GVRVQVEDTTKQERY----------GGEDYEMGETLESGTDAWN

Human-MCIP1 -------------------------------------------TTPSVVVHVCESDQEKE

ScRcn1 -----------------------------------------NGTFTLLKSKVGAITIDRC

:

MoCbpA DA------FMSPIDV-PKPILAHTARPPVELMHAA---

FfCbpA --------EVSPVEQ-SKPILAHTARPPVELMHHA---

NcCbpA ADGDIDMSPVSDSPR-TRPIFAHTARPPVELMHYA---

AfCbpA DE---DIEMMSPIDMSVRKLPPKTARPPVELMH-----

CnCbp1 PP----SQSGGMGTPGFGVKIMPTAMPPL---------

Human-MCIP1 EE------EEMERMRRPKPKIIQTRRPEYTPIHLS---

ScRcn1 PT-------NDGNGQMQLADHVKTAFPPKSIFDTDDDD

* *

**Fig. S3.** Clustal alignment of the CbpA proteins from different fungi and human MCIP1. While there is clear homology observed in the SP repeat motif (SPPxSPP; highlighted in yellow), the non-conservation of the ELHA and PxIxIT motifs (highlighted in red) may be noted. The phosphorylated residues in *A. fumigatus* CbpA are highlighted in green. MoCbpA-*Magnaporthe oryzae* CbpA; FfCnaA-*Fusarium fujikuroi* FfcbpA; NcCbpA-*Neurospora crassa* CbpA; AfcbpA-*Aspergillus fumigatus* CbpA; CnCbpA-*Cryptococcus neoformans* CbpA; Human-MCIP1; ScRcn1-*Saccharomyces cerevisiae* Rcn1.
