# Supplementary material for: Identification and mutational analyses of phosphorylation sites of the calcineurin-binding protein CbpA and the identification of domains required for calcineurin binding in Aspergillus fumigatus
Source: Front Microbiol. 2015 Mar 13;6:175. doi: 10.3389/fmicb.2015.00175 (PMC4358225; doi:10.3389/fmicb.2015.00175)
Supplement: Supplementary file 2 [file data_sheet_2.docx]

**Supplemental Table S1: List of primers used in this study**

| **Name** | **Sequence (5’-3’)** | **Direction** |
| --- | --- | --- |
| **Deletion of *cbpA***  CbpA-promo-KpnI-F  CbpA-term-HindIII-R  CbpA probe-F  CbpA probe-R | CGGGGTACCCTAGGATACTCCTGATAATATTAACAG GCAGAAGCTTAGCTAACGATGAAGCTGAACGAAC  GAACCTCCCTATAACGAG  TGCTTGCCACCGAGATAA | Forward  Reverse  Forward  Reverse |
| **CbpA-EGFP Expression**  CbpA-KpnI-F  CbpA-GFP-ovlp-R  CbpA-GFP-ovlp-F  GFP-NotI-R  CbpA-term-SbfI-F  CbpA-term-HindIII-R | CAGGGTACCATGGCGGACATCACAACGACATCA  cccgggggatccATGCATCAACTCAACCGGCGGGCGGAGTTGATGCATggatcccccgggctgcaggaattcAGCTGCGGCCGCtttacttgtacagctcgtccatgcc  GCACCTGCAGGTTGCTTTTCATCGATGGTCTTTTTCC  GCAGAAGCTTAGCTAACGATGAAGCTGAACGAAC | Forward  Reverse  Forward  Reverse  Forward  Reverse |
| **Mutations of *cbpA***  **S156A-S160A**  CbpA-S156A-S160A-F  CbpA-S156A-S160A-R  CbpA-KpnI-F  GFP-NotI-R  **S156D-S160D**  CbpA-S156D-S160D-F  CbpA-S156D-S160D-R  CbpA-KpnI-F  GFP-NotI-R  **S208A-S217A-S223A**  CbpA-S208A-F  CbpA-S208A-R  CbpA-S217A-S223A-F  CbpA-S217A-S223A-R  CbpA-KpnI-F  GFP-NotI-R | TTCATCGCTCCGCCCCCGGCCCCGCCA  TGGCGGGGCCGGGGGCGGAGCGATGAA  CAGGGTACCATGGCGGACATCACAACGACATCA  AGCTGCGGCCGCtttacttgtacagctcgtccatgcc  TTCATCGATCCGCCCCCGGACCCGCCA  TGGCGGGTCCGGGGGCGGATCGATGAA  CAGGGTACCATGGCGGACATCACAACGACATCA  AGCTGCGGCCGCtttacttgtacagctcgtccatgcc  GCCCATGGCGATGTCTGACGAG  CTCGTCAGACATCG**C**CATGGGC  GACGGGGGCCTGGCCCATCGCCATGGCAGGGCA  TGCCCTGCCATGGCGATGGGCCAGGCCCCCGTC  CAGGGTACCATGGCGGACATCACAACGACATCA  AGCTGCGGCCGCtttacttgtacagctcgtccatgcc | Forward  Reverse  Forward  Reverse  Forward  Reverse  Forward  Reverse  Forward  Reverse  Forward  Reverse  Forward  Reverse |
| ***cbpA* sequencing**  pUCGH-2033F  cbpA-371-seq-F  cbpA-561-seq-R | gcgttggccgattcatta  GCACAAAGATCTACTTCGGC  ATTGTTCCGTCTTGAGCTGC | Forward  Forward  Reverse |

| **CbpA Phosphorylated Peptides bound to CnaA (1)** | **Max**  **Mascot Score (2)** | **Position** | **Ascore Probability**  **(3)** |
| --- | --- | --- | --- |
| LFFI**S***PPP**S***PPHGWVMR | 42.5 | 156, 160 | 100% |
| TEQSAPVSGPVDPG**T***PM**S***MSDEKR | 108.5 | 205, 208 | >99%, <50% |
| TEQSAPVSGPVDPGTPM**S***MSDEKR | 49.5 | 208 | <50% |
| TEQSAPVSGPVDPG**T***PMSMSDEKR | 52.6 | 205 | >90% |
| TG**S***WPIAM**S***GQR | 50.8 | 217, 223 | <50%, >99% |
| TG**S***WPIAMSGQR | 47.0 | 217 | <50% |
| TGSWPIAM**S***GQR | 50.7 | 223 | >99% |

**Supplemental Table S2: Phosphorylated peptides in CbpA**

(1) Phosphorylated residues in the respective peptides are indicated by an asterisk. Phosphorylated residues in the phosphorylated domain I (PD-I) and phosphorylated domain II (PD-II) are colored red and green, respectively.

(2) Maximum ion scores were determined for each phosphorylated peptide across both individual samples.

(3) Probability of residue specific phosphorylation localization determined from <http://ascore.med.harvard.edu/>

**Supplemental Table S3: CbpA peptides identified to bind to CnaA by LC-MS/MS Analysis**

| **Experiment** | **Uniquely Identified Peptides (1)** | **Normalized Total Spectral Counts (2)** |
| --- | --- | --- |
| **Experiment 1** | SQIESIAPLNSFSPLPSLR  IYFGEPTPLLDEGRPK  TEQSAPVSGPVDPGTPMSMSDEKR TGSWPIAMSGQR | **4** |
| **Experiment 2** | SQIESIAPLNSFSPLPSLR  LLDGQSLLNR  IYFGEPTPLLDEGRPK  TEQSAPVSGPVDPGTPMSMSDEKR TGSWPIAMSGQR  EVHASDLAQALAQL | **7** |

(1) Total number of unique peptide sequences identified to CbpA within the corresponding LC-MS/MS analysis

(2) Normalized spectral counts reported from Scaffold following adjusting the sum of the selected quantitative value for all proteins in the list within each MS sample to a common value: the average of the sums of all MS samples present in the experiment.
