# Supplementary material for: Identification and mutational analyses of phosphorylation sites of the calcineurin-binding protein CbpA and the identification of domains required for calcineurin binding in Aspergillus fumigatus
Source: Front Microbiol. 2015 Mar 13;6:175. doi: 10.3389/fmicb.2015.00175 (PMC4358225; doi:10.3389/fmicb.2015.00175)
Supplement: Supplementary file 3 [file data_sheet_3.docx]

**Supplemental Materials and Methods**

**Phosphopeptide enrichment and LC-MS/MS analysis**

For the phosphopeptide analysis, samples were lyophilized to dryness using vacuum centrifugation and resuspended in 65 µl 80% acetonitrile, 1% TFA. Peptides were subjected to phosphopeptide enrichment using a 10 µl Protea Biosciences TiO2 Spin Tip and subsequently washed with 80% acetonitrile, 1% TFA. Peptides were eluted in 50 µl 20% acetonitrile, 5% aqueous ammonia, pH 10.5, and then acidified to pH 2.5 with formic acid prior to lyophilization to dryness.

Samples were resuspended in 10 µl 2% acetonitrile, 0.1% formic acid and subjected to chromatographic separation on a Waters NanoAquity UPLC equipped with a 1.7 µm BEH C18 75 µm I.D. x250 mm reversed-phase column. Phosphopeptide enriched samples were additionally supplemented with 10 mM citric acid. The mobile phase consisted of (A) 0.1% formic acid in water and (B) 0.1% formic acid in acetonitrile. Following a 5 µl injection, peptides were trapped for 5 min on a 5 µm Symmetry C18 180 lm I.D. x20 mm column at 5 µl/min in 99.9% A. A linear elution gradient of 5% B to 40% B was performed over 90 min at 400 nl/min.

The analytical column was connected to a fused silica PicoTip emitter (New Objective, Cambridge, MA) with a 10 µm tip orifice. Non-phosphopeptide enriched samples were analyzed on a Synapt QToF mass spectrometer operating in a data-dependent mode of acquisition with a precursor MS scan from m/z 400-2000 followed by three MS/MS scans at a CID energy of 30% and a dynamic exclusion of 30 s. Phosphopeptide enriched samples were analyzed on an Orbitrap XL mass spectrometer with a precursor MS scan from m/z 300–2000 with a target AGC setting of 1e6 ions. In a data-dependent mode of acquisition, MS/MS spectra of the five most abundant precursor ions were with a CID energy setting of 27 and a dynamic exclusion of 20 s was employed for previously fragmented precursor ions.

Raw LC-MS/MS data files were processed in Mascot distiller (Matrix Science) and then submitted to independent Mascot searches (Matrix Science) against a custom NCBI_Aspergillus database containing both forward and reverse entries of each protein. Search tolerances were 20 ppm or 10 ppm for precursor ions and 0.04 Da or 0.8 Da for product ions using trypsin specificity with up to two missed cleavages for non-phosphopeptide enriched or phosphopeptide enriched data, respectively. Carbamidomethylation (+57.0214 Da on C) was set as a fixed modification, whereas oxidation (+15.9949 Da on M), deamidation (+0.98 Da on NQ), and phosphorylation (+79.98 Da on STY) were allowed. All searched spectra were imported into Scaffold (v4.0, Proteome Software) and scoring thresholds were set to achieve a protein false discovery rate of < 1.0% using the PeptideProphet algorithm.
